# Comment on “Probiotics Can Further Reduce Waist Circumference in Adults with Morbid Obesity after Bariatric Surgery: A Systematic Review and Meta-Analysis of Randomized Controlled Trials”

**DOI:** 10.1155/2023/9769310

**Published:** 2023-09-26

**Authors:** Larry E. Miller, Ruemon Bhattacharyya

**Affiliations:** Department of Biostatistics, Miller Scientific, Johnson City, TN, USA

The meta-analysis by Zhang et al. [1] titled “Probiotics Can Further Reduce Waist Circumference in Adults with Morbid Obesity after Bariatric Surgery: A Systematic Review and Meta-Analysis of Randomized Controlled Trials” evaluated randomized controlled trials comparing the effect of probiotics versus placebo on anthropometric and biochemical changes in morbidly obese adults undergoing bariatric surgery. Unfortunately, there is a serious methodological error that nullifies the main conclusion stated in the manuscript title.

A primary conclusion of the meta-analysis was that probiotic use may further reduce waist circumference 12 months following bariatric surgery. The data used to arrive at this conclusion are reported in Figure 6(b). In calculating the placebo response from the study of Sherf-Dagan et al. [2], the authors erroneously used the 6-month data instead of the 12-month data. From Table 2 in this paper, the authors used a value of −24.5 ± 6.7 cm (the 6-month change value) for the meta-analysis instead of −29.7 ± 8.1 cm (the correct 12-month change value). This error fundamentally changes the conclusion of the paper. Based on the meta-analysis findings, the authors concluded that after 12 months, the mean difference in the waist circumference change was −4.2 cm (*p*=0.007), statistically favoring the probiotic group. However, when the correct 12-month data are used and the meta-analysis repeated, there is no difference between the probiotic and placebo groups (mean difference −0.7 cm, *p*=0.68). Ultimately, the meta-analysis results support the conclusion that probiotics offer no benefit to morbidly obese adults after bariatric surgery.
